# Supplementary material for: Identification and functional analysis of non-coding regulatory small RNA FenSr3 in Bacillus amyloliquefaciens LPB-18
Source: PeerJ. 2023 May 15;11:e15236. doi: 10.7717/peerj.15236 (PMC10194069; doi:10.7717/peerj.15236)
Supplement: Supplemental Information 4 [file peerj-11-15236-s004.zip › KO/CK-vs-T1_map/map05321.html]

KEGG PATHWAY: Inflammatory bowel disease (IBD) - Reference pathway


|  |  |
| --- | --- |
| **Inflammatory bowel disease (IBD) - Reference pathway** |  |

[
Pathway menu
| Organism menu
| Pathway entry
| Show description
| User data mapping
]

Inflammatory bowel disease (IBD), which includes Crohn disease (CD) and ulcerative colitis (UC), is characterized by chronic inflammation of the gastrointestinal tract due to environmental and genetic factors, infectious microbes, and the dysregulated immune system. Although many environmental factors (for example, geographic locations, smoking, etc.) affect the development of IBD, the most crucial might be the luminal (external) environment of the epithelial cells. There are pathogens that are found in increasing frequency in IBD. The microbial components such as flagellin, peptidoglycan, and lipopolysaccharide are recognized by receptors such as toll-like receptors (TLRs) and nucleotide-binding oligomerization domain (NOD) proteins, and also by antigen-presenting cells (APCs) in genetically susceptible host. The TLR recognition triggers the activation of NF-kappaB, leading to an inflammatory response. APC-expressed gene NOD2 has been associated with Crohn disease. In case of mutations of NOD2, negative regulation of IL-12 production is reduced with the stimulation of muramyl dipeptide (MDP), leading to CD. In addition, the APC mediates the differentiation of naive T cells into effector T cells (Th1, Th17, Th2) and natural killer T (NKT) cells. Th1 and Th17 cells produce high levels of IFN-gamma and IL-17, -22, respectively, both of which promote CD. In contrast, Th2 cells produce IL-4, -5, -10, which together with IL-13 from NKT induce UC.

|  |  |  |
| --- | --- | --- |
| Reference pathway | 184% 150% 122% 100% 82% 67% 55% | 图片下载 |
